# Corneal endothelial morphology of healthy myopic Malaysian children of Chinese ethnicity aged 8-9 years and its association with axial length

**DOI:** 10.12688/f1000research.110560.2

**Published:** 2022-09-07

**Authors:** Mohidin Norhani, Yu Chen Low, Mohd-Ali Bariah, Mohamad Shahimin Mizhanim, Arif Norlaili

**Affiliations:** 1 Centre of Optometry, Faculty of Health Science, Universiti Teknologi MARA, Puncak Alam Campus, Selangor, 42300, Malaysia; 2 Optometry and Vision Science Program, Research Centre for Community Health, Faculty of Health Science, Universiti Kebangsaan Malaysia, Kuala Lumpur, 50300, Malaysia

**Keywords:** endothelial cells density, myopia, axial length, myopic children

## Abstract

**Background**: This is a cross-sectional study to further understand the effects of axial length elongation on the corneal endothelial cell’s morphology in myopic children. Abnormal changes in the morphology of corneal endothelium are indicators of corneal stress or instability which could be linked to myopia.

**Methods: **111 school children comprising of 37 emmetropes, 37 mild myopes and 37 moderate myopes aged 8-9 years old were recruited. Visual acuity was measured using the LogMar chart, cycloplegic refraction was determined using an open-field autorefractor (Grand Seiko WAM-5100, Hiroshima, Japan) and refined using subjective refraction. Morphology of corneal endothelial cells [endothelial cell density, coefficient of variation, hexagonality and central corneal thickness] was evaluated using a non-contact specular microscope (Topcon SP-2000P). Axial length was measured with A-scan ultrasound biometry (PacScan Plus, Sonomed Escalon, NY). The correlation between morphology of corneal endothelial cells and axial length were assessed using Pearson Correlation and Linear regression analysis.

**Results:** There was no significant difference in corneal endothelial cells and axial length between gender (p>0.05). Significant reduction in endothelial cells density and hexagonality and increased coefficient of variation was found in eyes of higher myopic power which had longer axial when compared to emmetropes (p<0.001). Except for central corneal thickness, all corneal endothelial cells parameters correlated significantly with axial length (p<0.05). For every 1mm increase in axial length, endothelial cells density decreased by 73.27cells/mm ^2^, hexagonality decreased by 2.32% and coefficient of variation increased by 1.75%.

**Conclusions**: There were significant changes in morphology of cornea endothelial cells in young moderate myopic children of Chinese ethnicity at 8-9 years of age. This result provides normative data for Malaysian children of Chinese ethnicity that can be used for comparison and reference for clinical procedures, thereby facilitating decision-making with respect to interventions for myopia control, especially in prescribing contact lens for children.

## Introduction

The human corneal endothelial layer is made of a monolayer hexagonal shape cells arranged in a mosaic pattern. ^1^ Its morphology status is usually described in three aspects: endothelial cell density (ECD), which is the number of corneal endothelial cells per square millimetre (mm ^2^); coefficient of variation (COV), which is the mean cell area divided by the standard deviation (SD) of cell area; and percentage of six-sided hexagonal cells (% HEX). The CEC cells are responsible for regulating fluid and ion transport between aqueous humor and corneal stroma to maintain corneal thickness and transparency. ^2^ Unlike the epithelial cells, CEC are incapable of mitosis thus the number of cells diminish over time due to factors such as age, surgery, and trauma. ^3^^,^^4^ CEC have also been reported to be affected in myopia particularly myopia of high refractive power < -6.0D. ^5^^–^^7^

The prevalence of myopia onset in East Asian countries is increasing at an alarming rate at much younger ages. ^8^ Typical development of myopia begins during school-aged (8–14) years and continue to progress throughout teenage years until stabilization of the refractive error ^9^^,^^10^ Since the onset is occurring at much younger age there is a chance that it will progress into high myopia which may lead to visual impairment and possible blindness.

As myopia progresses, the eyeball elongates. This changes the whole dimension of the eye including anterior and posterior segment. These ocular changes include axial elongation, deepening of the anterior chamber and vitreous depth, thinning of the retina and sclera. ^11^ Progressive increase in axial length may result in posterior staphyloma, consequences of which include myopic maculopathy and myopic choroidal neovascularisation that eventually may lead to irreversible sight threatening complications. ^12^ While the cornea endothelium is notable for its absence of mitotic activity after birth, it is evident that flattening of CEC take place to compensate and spread over the enlarged area to form a continuous layer on the inner surface of the cornea. Subsequently, a reduced ECD is expected. ^13^^–^^16^ However, not many studies have reported on the effects of myopia on the cornea particularly the corneal endothelial cell (CEC). ^12^

Changes in the corneal endothelial cells (CEC) in myopes with high refractive power have been reported. ^5^^,^^6^ Sheng & Bullimore ^5^ studied the effects of degree of myopia on CEC and found that increased of COV & decreased in HEX is significantly correlated with refractive error. Chang *et al.* ^6^ studied 216 Asian subjects with a mean age of 22.2 years and found that corneal ECD was significantly lower in more myopic eyes. Urban *et al.* ^7^ conducted a study in myopic participants aged 13-18 years old to determine whether myopia could have an influence on CEC and found that ECD decreased in eyes with high myopia. Delshad and Chun ^17^ reported that moderate myopic eyes (power -4.25±0.78D) showed lower ECD values compared to low myopic eyes (power -1.51±-2.75D) in Malaysian of Chinese ethnicity with a mean age of 21.6 years. A study conducted in Spain analysed n=255 subjects with mean age of 38.6±11.8 years, 40.7±12.2 years, and 39.2±10.5 years for emmetropic, myopic and hyperopic subjects respectively. In that study, authors found significant changes between age and ECD but not spherical equivalent refractive error (SER) and ECD. ^18^ However ECD changes in the anterior segment during myopia progression are less well documented in children population. ^7^

High myopia is also associated with increase in axial length of the eye. ^12^ Chang *et al.* ^6^ found that CEC reduced with longer AL in a group of Chinese myopic young adults with a mean age of 22.2±4.2 years. Panjwani and Daigavane ^19^ examine ECD in 20 emmetropic subjects and 20 axial myopic subjects and reported a significant correlation between ECD and AL among emmetropes and myopes aged 15-49 years (n=80 eyes) and ECD values were significantly decreased in the axial myopia group with AL above 24 mm. In a study of elderly patient with mean age 75.7 years old and mean AL 23.16±0.93 mm, (range 21.71-27.12 mm), found a significant negative correlation between ECD and AL. ^20^ Thus, the authors suggested that eyes with longer axial length values are associated with a lower central ECD.

Although several studies have investigated the changes of CEC morphology in healthy young adults of Chinese ethnicity, there is still a lack of information on CEC morphology in myopic school children aged 8-9 years. This is because CEC studies are not usually performed on children’s eyes. To the best of our knowledge, this is the first study that examined CEC changes in the eye as results of increasing myopia in young school children. Part of the study is to examine changes in the morphology of CEC and its association with AL in a sample of 8-9 years old Malaysian schoolchildren of Chinese ethnicity. The result of this study will extend our understanding on the effect of AL elongation on CEC in young myopic children. Malaysia is a multiracial country with predominantly Malays, Chinese and Indians in its population. This study focuses on children of Chinese ethnicity due to its higher prevalence of myopia compared to other races in Malaysia. ^21^

## Methods

This study was approved by the UKM research ethics committee (UKM PPI-800-1/1/5 JEP- 2017-422) and followed the tenets of the Declaration of Helsinki.

The inclusion criteria for this study were age 8-9 years old, spherical equivalent refractive error +0.50<SER<-5D, astigmatism less than ≤1.50D in both eyes, no anisometropia and best corrected vision acuity (BCVA) 0.0 log of minimal angle of resolution (log MAR) in each eye, birth weight of ≥ 2000g, no history of ocular or systemic disease, not on myopia treatment, nor using contact lenses.

This study was a cross-sectional study conducted to examine the morphology of corneal of endothelial cells in healthy Malaysian Chinese school children with emmetropia and mild to moderate myopia and its association with axial length. The participating children were students at primary schools in Kuala Lumpur, Malaysia. Participants were recruited through advertisements placed around the optometry clinic and by words of mouth. Written consent was obtained from parents and participants prior to data collection. Participants were first screened for eligibility based on the inclusion criteria. Sample size of 110 was derived from G power calculation using settings of one tailed, estimated correlation effect size of 0.235 using previous study, ^20^ 0.8 as power of test (1 – β), and significance level of 0.05 (α).

All participants underwent a complete eye examination, including evaluation of visual acuity for distance and near vision using logMar chart. One drop of local topical anesthetic (Proxymetacaine Hydrochloride 0.5%, Alcaine, Alcon,15 mL) followed by two drops of cycloplegic eye drop (Cyclopentolate Hydrochloride 1%, Cyclogyl, Alcon, 15 mL) at 5 minutes interval for each drop, used to paralyze the ciliary muscle and inhibit accommodation. The amplitude of accommodation was assessed using an RAF rule to ensure that accommodation was paralyzed and cycloplegic refraction was conducted when pupil size achieves ≥5 mm. Cycloplegic refraction using open-field autorefractor (Grand Seiko WAM-5100, Hiroshima, Japan) and later refined using cross cylinder technique. Slit lamp (RIGHTON MW50D LED, Tokyo, Japan) was used to examine the anterior eye structure, and corneal topographer (TMS-4N; Tomey, USA) was used to map the curvature of the corneal surface. Axial length was measured using A-scan ultrasound biometry (PacScan Plus, Sonomed Escalon, New York, USA). The A-Scan result was calculated by a single continuous beep which automatically records the mean of five measurements with the standard deviation of <0.10 mm.

Central CEC morphologies [ECD, HEX, COV and Central Corneal Thickness (CCT)] were evaluated by a single examiner using a non-contact specular microscope (SP-3000P; Topcon, Tokyo, Japan). The accuracy and reliability of this model have been reported in several comparison studies. ^22^^–^^24^ All measurements were carried out in automatic mode under room illumination between 480–600 lux at 9-11am in the morning to avoid diurnal variation. Three microphotographs were performed for every eye (the difference of ECD and CCT values did not exceed±5%), and the average value was calculated.

To achieve the most accurate measurement, 50 adjacent CEC were manually selected on the specular photomicrograph of a 0.5 × 0.25 mm section of endothelial surface. Following that, the device performed an automatic analysis of the selected area and calculated the average number of cells per 1 mm ^2^ and the CCT in μm. The microscope then provided a histogram determining the endothelial cells density (ECD, cell/mm2), population size and specified the minimum, maximum, and average cell size of the selected area. The pleomorphism of endothelial cells was also evaluated, indicating the coefficient of variation in percentage (% COV), percentage of cells’ hexagonality (% HEX). The procedure was repeated three times and average values noted.

The data obtained in this study was analysed using statistical software Statistical Package for the Social Sciences (SPSS Inc., Chicago, version 21.0), at a significance threshold of 5% (p<0.05). Kolmogorov-Smirnov test for normality was conducted on ECD (KS=0.041, df=111, p value=0.200) and AL (KS=0.052, df=111, p value=0.200), both showed normal distribution. Parametric tests were then used for subsequent analysis.

There were no significant between two eyes and the results showed that right eye and left eye were highly correlated. Hence, only the data from right eye was reported and analysed to avoid confounding effect from inter-ocular correlation. ^25^^,^^26^ Student’s t-test was initially performed to evaluate differences between gender in CEC and AL. For purpose of comparison in CEC and CCT between the different refractive groups, participants were divided into three groups following classification noted by Baird *et al.*, ^27^ namely i) emmetropia (SER>−0.5D), ii) mild myopia (−3.00D < SER ≤ -0.50D) and iii) moderate myopia (-5.00D < SER ≤ -3.00D). Participants were also divided based on axial length. Mean of AL for each refractive group was calculated and three groups were arbitrarily divided as follows: i) AL <23 mm, ii) AL between 23-24 mm and iii) AL >24 mm. One way ANOVA F test was employed to analyse the differences of CEC morphology between the three groups. Pearson’s correlation and Simple linear regression test were performed to determine the relationship between AL and CEC morphology.

## Results

A total of 111 Malaysian children (57 males/54 females) of Chinese ethnicity were examined. Demographic profile for all participants is depicted in Table 1. The mean age of all participants was 8±0 years old (range, 8 to 9 years old). Mean cycloplegic SER was -2.01±1.73D (range -2.34 to -1.68). There was no significant difference between genders for any factors including the mean SER, age, VA, ECD, HEX, COV, CCT and AL.

**Table 1.** Participant demographic profile.

	All participants (n=111)	Male (n=57)	Female (n=54)	*p value (males vs females)
Age (years)	9±1	9±0	8±1	0.308
BCVA	0±0.06	0±0.06	0±0.07	0.843
Spherical Equivalent (D)	-2.01±1.73	-1.95±1.7	-2.08±1.79	0.687
AL (mm)	23.67±1.13	23.79±1.14	23.54±1.13	0.255
K cornea (D)	43.34±1.40	43.16±1.37	43.52±1.42	0.255
CCT (μm)	527.01±26.17	527.28±27.46	526.72±24.99	0.910
ECD (/mm ^2^)	3232.5±239.66	3192.12±243.29	3275.12±230.36	0.068
HEX (%)	58.39±10.36	58.98±10.3	57.76±10.49	0.537
COV ^a^	41.67±8.51	41.22±8.48	42.15±8.59	0.568

Data are expressed as means±standard deviation, D *dioptre*, AL *axial length*, CCT *central corneal thickness*, ECD *endothelial cell density*, HEX *hexagonality*, COV *coefficient of variation*.

* T test: [Age: t(109)=1.024, p>0.05], [BCVA: t(109)=-0.199, p>0.05], [SE: t(109)=0.404, p>0.05], [AL: t(109)=1.143, p>0.05], [K cornea: t(109)=-1.371, p>0.05], [CCT: t(109)=0.113, p>0.05], [ECD: t(109)=-1.844, p>0.05], [HEX: t(109)=0.62, p>0.05], [COV: t(109)=-0.573, p>0.05].

The mean SER of emmetrope, mild and moderate myope were 0.05±0.24D, -2.06±0.58D and -4.01±0.57D respectively. ANOVA showed significant differences in AL and CEC (ECD, HEX, COV) parameters between the three refractive groups but not for CCT and Cornea curvature (K), as shown in Table 2.

**Table 2.** AL and CEC parameters (ECD, HEX, COV, and CCT) between different refractive groups.

	All participants	Emmetrope (n=37) SER>−0.5	Low myope (n=37) −3.00 < SER ≤ -0.50	Moderate myope (n=37): -5.00 < SER ≤ -3.00	*p value
SER (D)	-2.01±1.73	0.05±0.24	-2.06±0.58	-4.01±0.57	0.001 *
AL (mm)	23.67±1.13	22.7±0.8	23.61±0.78	24.69±0.78	0.001 *
K cornea (D)	43.34±1.4	43.78±1.22	43.21±1.37	43.02±1.53	0.053
ECD(/mm ^2^)	3232.5±239.66	3344.83±206	3251.43±219.88	3101.24±231.67	0.001 *
HEX (%)	58.39±10.36	64.08±7.03	56.3±10.86	54.78±10.46	0.001 *
COV	41.67±8.51	36.58±6.6	43.81±8.37	44.64±8.19	0.001 *
CCT (μm)	527.01±26.17	531.08±22.63	520.88±24.51	529.05±30.32	0.208

Data are expressed as means±standard deviation. SER *spherical equivalent refraction*, D *dioptre*, ECD *endothelial cell density,* HEX *hexagonality*, COV *coefficient of variation* CCT *central corneal thickness*, AL *axial length*.

* ANOVA: [SER: F(2, 108)=641.062, p<0.05], [A: F(2, 108)=59.7, p<0.05], [K: F(2, 108)=3.023, p>0.05], [ECD: F(2, 108)=11.605, p<0.05], [HEX: F(2, 108)=9.978, p<0.05], [COV: F(2, 108)=12.084, p<0.05], [CCT: F(2, 108)=1.592, p>0.05].

Post hoc analysis showed ECD values in moderate myopic group (mean = 3101.24±231.67 cells/mm ^2^) was significantly lower than the mild myopic (mean = 3251.43±219.88 cells/mm ^2^) and emmetropic group (mean 3344.83±206 cells/mm ^2^), (p<0.001). There is no significant differences (p=0.164) in ECD in values between emmetropic and mild myopic group.

The mild myopic (56.3±10.86 %) and moderate myopic group (54.78±10.46 %) had significantly lower HEX values compared to emmetropic group (64.08±7.03 %, p<0.001). There was no significant differences (p=0.777) in HEX values between mild myopic group and moderate myopic group.

COV value in mild myopic (43.81±8.37%) and moderate myopic groups (44.64±8.19%) were significantly higher than the emmetropic group (36.58±6.6%, p<0.001). Post hoc also showed no significant differences (p=0.892) in COV values between mild myopic and moderate myopic group.

The results also showed that moderate myopic group has the longest AL, (mean=24.69±0.78mm), followed by mild myopic (mean=23.61± 0.78mm) and emmetropic group (22.7±0.8). The distribution of participants according to AL is shown in Table 3.

**Table 3.** CEC parameters (ECD, HEX, COV, and CCT) between different AL groups.

	All participants	< 23 mm (n=33)	23-24 mm (n=35)	> 24 mm (n=43)	*p value
SER (D)	-2.01±1.73	-0.39±0.83	-1.73±1.45	-3.48±1.15	0.001 *
AL	23.67±1.13	22.32±0.43	23.51±0.31	24.82±0.58	0.001 *
K	43.34±1.4	43.75±1.46	43.72±1.06	42.71±1.4	0.001
ECD (/mm ^2^)	3232.5±239.66	3325.51±227.13	3267.65±203.67	3132.51±243.89	0.001 *
HEX (%)	58.39±10.36	60.88±9.66	58.51±10.24	56.37±10.77	0.171
COV	41.67±8.51	39.98±7.99	41.74±9.81	42.92±7.69	0.331
CCT (μm)	527.01±26.17	529.32±20.8	518.77±24.91	531.93±29.6	0.071

Data are expressed as means±standard deviation.

AL *axial length*, ECD *endothelial cell density*, HEX *hexagonality*, COV *coefficient of variation*, CCT *central corneal thickness*.

* ANOVA: [SER: F(2, 108)=66.474, p<0.05], [AL: F(2, 108)=272.442, p<0.05], [K: F(2, 108)=7.856, p<0.05], [ECD: F(2, 108)=7.368, p<0.05], [HEX: F(2, 108)=1.796, p>0.05], [COV: F(2, 108)=1.117, p>0.05], [CCT: F(2, 108)=2.705, p>0.05].

A significant difference in mean ECD (p<0.001) was observed between the three AL groups. Post hoc test further showed that participants with AL greater than 24 mm had significantly lower ECD readings (3132.51±243.89) compared to 23 – 24 mm group (3267.65±203.67), and < 23 mm group (3325.51±227.13), (p<0.001). Significant different in K cornea reading was found between AL groups, in which group with AL > 24 mm had significantly flatter K cornea reading (p=0.001) compared to those with AL 24 mm and below. No significant differences in HEX, COV, and CCT found between the AL groups.

**Figure 1.**
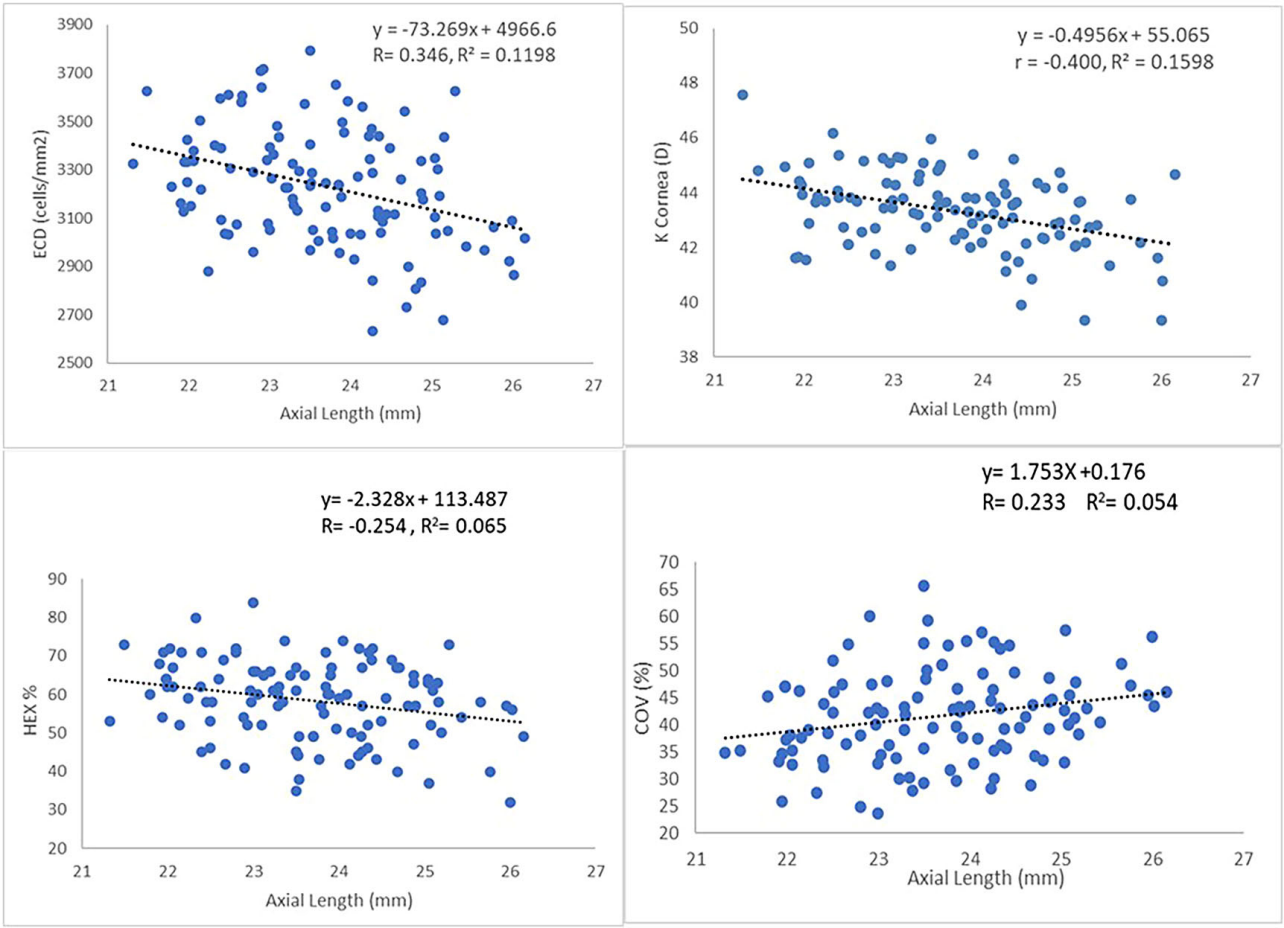
Relationship between ECD, HEX, COV, K and AL. ECD *endothelial cell density*, HEX *hexagonality*, COV *coefficient of variation*, K *cornea curvature and* AL *axial length.*

Except for CCT, significant correlation was found between AL and all CEC parameters ( Table 4). The results showed AL correlates significantly with ECD (r=-0.346, p<0.001), HEX (r=-0.254, p<0.007) and COV (r=-0.233, p<0.014) and cornea curvature (r=-0.400, p<0.001). Pearson correlation showed for every 1mm increase in AL, there was a significant reduction of 73.27 cells/mm ^2^ in ECD (R ^2^ = 0.12), a reduction of 2.32 per cent in HEX (R ^2^ = 0.065), increase of 1.753 per cent in COV (R ^2^ = 0.054), and -0.4956 flattening of K cornea (D) ( Figure 1).

**Table 4.** Relationship between AL and CEC (corneal endothelial cell) parameters.

	X1	X2	r	p value
Correlation	AL	ECD	-0.346	0.001
Correlation	AL	HEX	-0.254	0.007
Correlation	AL	COV	-0.233	0.014
Correlation	AL	SER	-0.781	0.001
Correlation	AL	K- cornea	-0.400	0.001
Correlation	AL	CCT	0.055	0.566
Correlation	K- cornea	CCT	-0.054	0.573

AL *axial length*, ECD *endothelial cell density*, HEX *hexagonality*, COV *coefficient of variation*, SER spherical equivalent refraction, K cornea *mean corneal curvatures*, CCT *central corneal thickness*.

r=Pearson’s correlation coefficient. p=two tailed statistical significance by Pearson’s correlation test.

Longer AL had lesser ECD (r=-0.346**, p<0.05), longer AL had lesser HEX (r=-0.254**, p<0.05), longer AL had increased COV (r=-0.233*, p<0.05), longer AL had more negative SER (r=-0.781**, p<0.05), longer Al had had flatter cornea curvature (r=-0.400**, p<0.05). However, CCT correlated neither with AL (r=0.055, p=0.566) nor with corneal curvature (r=−0.054, p=0.573).

* P<0.05.

** P<0.01.

## Discussion

In the present study, insignificant difference in CEC morphology was observed between genders, which support findings from other studies in adults ^19^^,^^28^ and children ^14^^,^^29^ However in a study conducted in Japan, ^30^ it was shown that female participants had lower ECD values, increased COV and decreased HEX when compared to males. Their study was carried out among older adults with mean age 61.8± 10.2 years with high myopia (SER ≤ -6D) and history of contact lens wear among its female participants. All these could contribute to the differences in findings since other studies have shown that increase in age, ^29^ presence high myopia, ^17^^,^^18^ contact lens wear ^31^ could affect CEC morphology.

With regards to CEC morphology, this study found a statistically significant difference in children with different refractive errors. The results showed a reduction in ECD values in the moderate myopic group compared to mild myopic and emmetropic groups. There was also a decrease in HEX and increase of COV in mild and moderate myopic group compared to the emmetropic group. A study by Delshad & Chun ^17^ among Malaysian Chinese adults 18-24 years old, also found relatively more myopic eyes have reduced ECD and HEX compared to mild myopic eyes. Chang *et al.* ^6^ in Taiwan also reported ECD decreased in eyes with increased myopia among participants with a mean age of 22 years. CEC values in young myopes (age 8-9 years old) have not been reported. CEC parameters reduced in adults wearing contact lenses and it was shown to be associated with pleomorphism and polymegathism. ^31^ It is possible that changes in CEC parameters that occur in myopic children 8-9 years old with moderate myopic power may indicate that pleomorphism and polymegathism may have already occurred. These factors need to be validated in future studies and could be considered in contact lens related myopia control management in very young myopes.

It is fairly established that increase in myopia is likely to be associated with increase in axial length. ^32^^,^^33^ When our participants were grouped and analysed according to differences in axial length the results showed that participants with longer AL have reduced ECD. The same trend has been reported whereby lower ECD values were associated with longer eyes. ^6^^,^^17^^,^^20^ Chang *et al.*, ^6^ suggested if the limbal dimension did not change, elongation of the axial length led to deepening of anterior chambers and an increase in endothelial surface area. The authors revealed that due to lack of mitotic activity of the corneal endothelium after birth, flattening of the endothelial cells was expected in order to cover the enlarged surface and these changes reduce ECD. Although ECD reduction has occurred among the myopic group with longest AL (>24 mm), HEX and COV was not found to be significantly different in subjects with longer AL in this study. This indicate that pleomorphism and polymegathism changes in myopic eyes are mainly affected by the changes in anterior cornea curvature but not posterior AL elongation, which agrees with Urban et al., who reported that ECD decreased in eyes with high myopia but does not correlate either with CCT nor with AL in myopic children. ^7^

The effect of myopia on CCT has reported conflicting results. In this study, no significant association between CCT and degree of myopia was observed. This finding agreed with a study conducted among Singaporean children ^34^ whereby CCT was not associated with refractive error. Similarly, Cho and Lam ^35^ found that central corneal thickness decreased with increasing age but not with refractive error or corneal curvatures. Chen *et al.* ^36^ also found that there is no statistically significant association between CCT and refractive error in their Chinese adult myopic population. The authors concluded that the cornea did not thin in the same way as the sclera in myopic eyes. The stretching of the globe was likely to be restricted only to the peripheral globe and did not influence the central CCT to a measurable degree. Several studies have also reported that CCT did not correlate with degree of myopia ^36^^–^^40^ In contrast, some studies found that the cornea tend to be thinner in highly myopic eyes. ^41^ The wide discrepancy could be attributed to the differences in age groups studied, smaller sample sizes, influence of diurnal variation, and use of different pachymeters with variable reproducibility. ^42^

This is the first study that evaluates a narrow age-range of young school children of Chinese ethnicity in Malaysia. Furthermore, not many studies have reported on ECD and morphology changes in the Chinese population. This study was carried out in Kuala Lumpur that has relatively high prevalence of myopia in the Chinese student population ^21^ due to urbanized settings along with the influence of environmental factors such as near work and outdoor time, ^43^^,^^44^ therefore it was particularly interesting to study the association of ECD and AL in this population. Increased knowledge of the changes in CEC morphology in myopic population is needed especially when the prevalence of myopia in Chinese school-aged children has exceeded 90%. ^43^

As this report presents the baseline data of the myopic children who participate in our longitudinal study to determine the cornea morphologies before and after Ortho-keratology lens treatment, the major limitations of this study were its cross-sectional nature and the small number of children with narrow age range that were examined. Hence, longitudinal study following the same participants into adulthood could refine our understanding of CEC morphology changes with AL elongation in myopia progression. Despite these limitations, we believe that the strengths of this study, which included examination of a homogenous population in a concise age group, could provide a referential data for practitioners to compare emmetropic and myopic patients in management of children population. We also hope that our results could be a future reference when practitioners decide whether to prescribe contact lens related myopia control intervention based on the CEC health.

## Conclusion

In conclusion, our study found significant changes in CEC morphology as myopic power and axial length increased. With the exception of CCT, all the CEC parameters examined were found to be significantly correlated to AL. The results imply that CEC of myopes even of moderate power are susceptible to mechanical stress. We also described a normative ECD data in Malaysian Chinese school children 8-9 years of age. Our results could serve as a useful baseline for future reference when practitioners decide whether to prescribe contact lens related myopia control intervention based on the CEC health.

## Data availability

### Underlying data

Dryad: Corneal endothelial morphology of healthy myopic Malaysian children of Chinese ethnicity aged 8-9 years and its association with axial length, https://doi.org/10.5061/dryad.hhmgqnkjc.

This project contains the following underlying data:

- F1000_ECD_raw_data_submitted.xlsx
- README.txt (Data legend)

Data are available under the terms of the Creative Commons Attribution 4.0 International license (CC-BY 4.0).
