# Supplementary material for: Corneal endothelial morphology of healthy myopic Malaysian children of Chinese ethnicity aged 8-9 years and its association with axial length
Source: F1000Res. 2022 Sep 7;11:339. Originally published 2022 Mar 21. [Version 2] doi: 10.12688/f1000research.110560.2 (PMC9459173; doi:10.12688/f1000research.110560.2)
Supplement: Peer review history

# Peer review: Reviewer response for version 2

**DOI:** 10.5256/f1000research.137370.r149711

**Published:** 2022-09-08

**Authors:** Jeffrey Tsz-wing Leung

**Affiliations:** 1 School of Optometry, The Hong Kong Polytechnic University, Hong Kong, China

The author has sufficiently addressed all comments.

Is the work clearly and accurately presented and does it cite the current literature?

Yes

If applicable, is the statistical analysis and its interpretation appropriate?

Yes

Are all the source data underlying the results available to ensure full reproducibility?

Yes

Is the study design appropriate and is the work technically sound?

Yes

Are the conclusions drawn adequately supported by the results?

Partly

Are sufficient details of methods and analysis provided to allow replication by others?

Partly

Reviewer Expertise:

Myopia, Astigmatism, Corneal shape

I confirm that I have read this submission and believe that I have an appropriate level of expertise to confirm that it is of an acceptable scientific standard.

# Peer review: Reviewer response for version 1

**DOI:** 10.5256/f1000research.122182.r144594

**Published:** 2022-08-01

**Authors:** Jeffrey Tsz-wing Leung

**Affiliations:** 1 School of Optometry, The Hong Kong Polytechnic University, Hong Kong, China

This study provides normative data on the morphology of corneal endothelial cells (CEC) in Chinese myopic children. The method is technically sound, and the statistical analyses are appropriate. However, the authors should explain more clearly the importance of this study. Although previous studies mainly focused only on adults, why is it essential to measure the morphology of CEC in children? Given that corneal endothelial cells are incapable of further mitosis, could we anticipate the data from young adults can already apply to children? If not, please explain. The authors should also indicate more explicitly how the data contribute to clinical decision-making on myopia control. Perhaps, clinicians should be more alert when prescribing myopia-control contact lenses. 

 Other comments:

- Introduction, 1st paragraph, last sentence: There is a missing full stop.
- Why have only 8-9 years old children been recruited but not a wider age range?
- Please explain why high myopes and high astigmats were excluded.
- Why did the authors assume one- instead of two-tailed statistics in sample size calculation?
- Please provide the details on the use of cycloplegia (e.g., cycloplegic agent, dosage, waiting time before refraction).
- Please explain why the experiment was conducted from 9-11 am.
- The authors only reported the right eye. Please also indicate if there were any differences between the eyes.
- Results should be written in the past tense. Please check.
- Table 3: Please indicate the sample size for each AL group.
- The authors need to explain why HEX and COV significantly differed among myopia groups but not in the AL groups.
- Table 4. Because p values were presented, the asterisks are not necessary.
- Discussion, 3rd paragraph: Because this study did not measure ACD, it is difficult to tell whether the results are attributable to the "enlarged surface" of the anterior segment of myopic eyes. The authors should be more conservative in their arguments.  

Is the work clearly and accurately presented and does it cite the current literature?

Yes

If applicable, is the statistical analysis and its interpretation appropriate?

Yes

Are all the source data underlying the results available to ensure full reproducibility?

Yes

Is the study design appropriate and is the work technically sound?

Yes

Are the conclusions drawn adequately supported by the results?

Partly

Are sufficient details of methods and analysis provided to allow replication by others?

Partly

Reviewer Expertise:

Myopia, Astigmatism, Corneal shape

I confirm that I have read this submission and believe that I have an appropriate level of expertise to confirm that it is of an acceptable scientific standard, however I have significant reservations, as outlined above.

# Response

**Published:** 2022-08-11

**Authors:** Yu Chen Low

**Affiliations:** Universiti Kebangsaan Malaysia, Malaysia

Dear Dr. Jeffrey Tsz-wing Leung,

 We would like to thank you for your careful and thorough reading of our submitted manuscript and for the thoughtful comments and constructive suggestions. These have greatly helped us to improve the quality of our manuscript. Our responses to your comments are shown below:

**Response to the Importance of this study:**

 This report presents the baseline data of the myopic children who participate in our longitudinal study to determine the cornea morphologies before and after Ortho-keratology lens treatment. It is essential to measure the morphology of CEC in children as previous studies have shown that changes in the morphology of corneal endothelium are indicators of corneal stress or instability which could be linked to myopia. However, studies in children population are scarce. Therefore, this study is to further understand the effects of axial length elongation on the corneal endothelial cell’s morphology in myopic children before the commencement of Ortho-K lens wear. We hope that our results could be a future reference when practitioners decide whether to prescribe contact lens related myopia control intervention based on the CEC health.

**RESPONSE TO OTHER COMMENTS**

1. Introduction, 1st paragraph, last sentence: There is a missing full stop.
  Response
  A full stop has been added.
2. Why have only 8-9 years old children been recruited but not a wider age range
  Response
  The age range 8-9 years old was used because previous Studies have reported that incidence and progression rates of myopia are high in children aged 7-9 years old (Saw et al., 2005; Tricard D et al., 2021). The findings also showed that annual change in spherical equivalence and AL peaked during the year of myopia onset. Furthermore, children younger than 8 years might not be able to understand and comply with the responsibilities of contact lens care and hygiene.
3. Please explain why high myopes and high astigmats were excluded.
  Response
  Following previous Ortho-K studies, Cho and Cheung (2012) recruited subjects with myopia -0.50 and -4.00D and astigmatism not more than 1.25D.
4. Why did the authors assume one- instead of two-tailed statistics in sample size calculation?
  Response
  This study focuses on finding the relationship/ correlation of axial length with CEC morphology. So based on a previous study, those relationships are only one directional. Thus, we made one directional hypothesis assumption when we estimate the sample size.
5. Please provide the details on the use of cycloplegia (e.g., cycloplegic agent, dosage, waiting time before refraction).
  Response
  One drop of local topical anesthetic (Proxymetacaine Hydrochloride 0.5%, Alcaine, Alcon,15mL) followed by two drops of cycloplegic eye drop (Cyclopentolate Hydrochloride 1%, Cyclogyl, Alcon, 15mL) at 5 minutes interval for each drop, used to paralyze the ciliary muscle and inhibit accommodation. The amplitude of accommodation was assessed using an RAF rule to ensure that accommodation was paralyzed and cycloplegic refraction was conducted when pupil size achieves ≥ 5mm.
6. Please explain why the experiment was conducted from 9-11 am
  Response
  (Read & Collins 2009) reported diurnal variation that occurs in the regional thickness and the shape of the anterior and posterior cornea. Therefore, to avoid any diurnal variation, all measurements were conducted from 9-11am.
7. The authors only reported the right eye. Please also indicate if there were any differences between the eyes.
  Response
  There were no significant between two eyes and the results showed that the right eye and left eye were highly correlated. Hence, only data from the right eye was included in the analysis to avoid the confounding effect of intercorrelation.
8. Results should be written in the past tense. Please check.
  Response
  Results have been changed to past tense.
9. Table 3: Please indicate the sample size for each AL group
  Response
  The sample size for each AL group have been indicated.
10. The authors need to explain why HEX and COV significantly differed among myopia groups but not in the AL groups.
  Response
  Line 235-240, explanation added.
11. Table 4. Because p values were presented, the asterisks are not necessary
  Response
  Asterisks were removed.
12. Discussion, 3rd paragraph: Because this study did not measure ACD, it is difficult to tell whether the results are attributable to the "enlarged surface" of the anterior segment of myopic eyes. The authors should be more conservative in their arguments
  Response
  Line 232-235, rephrased by citing the paper by Chang et al., 2001 who measured ACD in their study.

# Peer review: Reviewer response for version 1

**DOI:** 10.5256/f1000research.122182.r128138

**Published:** 2022-04-11

**Authors:** Mustafa Abdu

**Affiliations:** 1 Associate Professor of Optometry, Faculty of Optometry and Visual Sciences, Department of Contact Lenses, Al-Neelain University, Khartoum, Sudan

The study describes the association between the morphology of corneal endothelial cells and the changes in axial length of myopic eyes of children of Chinese ethnicity. In previous reports, it has been reported that the prevalence of myopia is the highest among this race, and I think that is why the authors chose this group of study.

 Overall, the study followed the correct methods in building their research and I support that findings will lead to reconsidering of myopia control strategies among this ethnic group.

 Table 1 title should be written as ' Participants' demographic and clinical profile'

Is the work clearly and accurately presented and does it cite the current literature?

Yes

If applicable, is the statistical analysis and its interpretation appropriate?

Yes

Are all the source data underlying the results available to ensure full reproducibility?

Yes

Is the study design appropriate and is the work technically sound?

Yes

Are the conclusions drawn adequately supported by the results?

Yes

Are sufficient details of methods and analysis provided to allow replication by others?

Yes

Reviewer Expertise:

Cornea, Contact Lenses, Myopia control and Keratoconus

I confirm that I have read this submission and believe that I have an appropriate level of expertise to confirm that it is of an acceptable scientific standard.

# Response

**Published:** 2022-04-26

**Authors:** Yu Chen Low

**Affiliations:** Universiti Kebangsaan Malaysia, Malaysia

Thank you Prof Dr.Mustafa for the peer review. We will revise and change our Table 1 title to  ' Participants' demographic and clinical profile'. Thank you again for your valuable time and comment. Much appreciated.
